# Effect of P-glycoprotein on flavopiridol sensitivity

**DOI:** 10.1054/bjoc.2000.1688

**Published:** 2001-05

**Authors:** S A Boerner, M E Tourne, S H Kaufmann, K C Bible

**Affiliations:** Divisions of Oncology Research andMedical Oncology, Mayo Clinic andDepartment of Molecular Pharmacology, Mayo Graduate School, Rochester, MN 55905, USA

**Keywords:** drug resistance, flavonoids, cyclin-dependent kinase, chemotherapy

## Abstract

Flavopiridol is the first potent inhibitor of cyclin-dependent kinases (CDKs) to enter clinical trials. Little is known about mechanisms of resistance to this agent. In order to determine whether P-glycoprotein (Pgp) might play a role in flavopiridol resistance, we examined flavopiridol sensitivity in a pair of Chinese hamster ovary cell lines differing with respect to level of Pgp expression. The IC _50_ s of flavopiridol in parental AuxB1 (lower Pgp) and colchicine-selected CH^R^C5 (higher Pgp) cells were 90.2 ± 6.6 nM and 117 ± 2.3 nM, respectively (*P*< 0.01), suggesting that Pgp might have a modest effect on flavopiridol action. Consistent with this hypothesis, pretreatment with either quinidine or verapamil (inhibitors of Pgp-mediated transport) sensitized CH^R^C5 cells to the antiproliferative effects of flavopiridol. Because of concern that colony forming assays might not accurately reflect cytotoxicity, we also examined flavopiridol-treated cells by trypan blue staining and flow cytometry. These assays confirmed that flavopiridol was less toxic to cells expressing higher levels of Pgp. Further experiments revealed that flavopiridol inhibited the binding of [^3^H]-azidopine to Pgp in isolated membrane vesicles, but only at high concentrations. Collectively, these results identify flavopiridol as a weak substrate for Pgp. © 2001 Cancer Research Campaign www.bjcancer.com


British Journal of Cancer (2001) 84(10), 1391–1396
© 2001 Cancer Research Campaign
DOI: 10.1054/ bjoc.2000.1688, available online at http://www.idealibrary.com on IDEAL®

### Effect of P-glycoprotein on flavopiridol sensitivity

**SA Boerner[1], ME Tourne[3], SH Kaufmann[1,3] and KC Bible[2,3]**

Divisions of [1]Oncology Research and [2]Medical Oncology, Mayo Clinic and [3]Department of Molecular Pharmacology, Mayo Graduate School, Rochester, MN 55905, USA

**Summary** Flavopiridol is the first potent inhibitor of cyclin-dependent kinases (CDKs) to enter clinical trials. Little is known about mechanisms of resistance to this agent. In order to determine whether P-glycoprotein (Pgp) might play a role in flavopiridol resistance, we examined flavopiridol sensitivity in a pair of Chinese hamster ovary cell lines differing with respect to level of Pgp expression. The $IC_{50}$s of flavopiridol in parental AuxB1 (lower Pgp) and colchicine-selected $CH^RC5$ (higher Pgp) cells were 90.2 ± 6.6 nM and 117 ± 2.3 nM, respectively ($P < 0.01$), suggesting that Pgp might have a modest effect on flavopiridol action. Consistent with this hypothesis, pretreatment with either quinidine or verapamil (inhibitors of Pgp-mediated transport) sensitized $CH^RC5$ cells to the antiproliferative effects of flavopiridol. Because of concern that colony forming assays might not accurately reflect cytotoxicity, we also examined flavopiridol-treated cells by trypan blue staining and flow cytometry. These assays confirmed that flavopiridol was less toxic to cells expressing higher levels of Pgp. Further experiments revealed that flavopiridol inhibited the binding of [$^3$H]-azidopine to Pgp in isolated membrane vesicles, but only at high concentrations. Collectively, these results identify flavopiridol as a weak substrate for Pgp. © 2001 Cancer Research Campaign http://www.bjcancer.com

**Keywords**: drug resistance; flavonoids; cyclin-dependent kinase; chemotherapy

Flavopiridol is the first potent cyclin-dependent kinase inhibitor to enter clinical trials as a potential anticancer agent (Sedlacek et al, 1996; Senderowicz et al, 1998; Wright et al, 1998). This agent inhibits proliferation (Kaur et al, 1992; Czech et al, 1995; Carlson et al, 1996; Sedlacek et al, 1996; Drees et al, 1997) and induces apoptosis in a variety of human cancer cells and cell lines (Bible and Kaufmann, 1996; de Azevedo et al, 1996; König et al, 1997; Schwartz et al, 1997; Arguello et al, 1998; Brüsselbach et al, 1998; Byrd et al, 1998; Parker et al, 1998; Patel et al, 1998). Based on its unique mechanism of action (Losiewicz et al, 1994), its ability to kill noncycling tumour cells (Bible and Kaufmann, 1996; Byrd et al, 1998) and its promising antitumour activity in xenograft models (Czech et al, 1995; Drees et al, 1997; Arguello et al, 1998; Patel et al, 1998), flavopiridol has entered phase I (Senderowicz et al, 1998) and phase II (Wright et al, 1998) testing as a single agent as well as phase I trials in combination with paclitaxel or cisplatin (Wright et al, 1998).

Despite the clinical interest in flavopiridol, relatively little is known about potential mechanisms of resistance to this agent. Comparison of paired cell lines expressing the P-glycoprotein (Pgp) multidrug transporter (K562 and K562R, 8226 and 8226/Dox40) reportedly failed to demonstrate Pgp-mediated alterations in drug sensitivity (Schlege et al, 1999). Likewise, examination of a flavopiridol-resistant ovarian cancer line revealed no cross-resistance to the Pgp substrates paclitaxel, etoposide or doxorubicin (Bible et al, 2000), suggesting that Pgp does not play a role in flavopiridol resistance of this particular cell line. As a hydrophobic natural product, however, flavopiridol resembles other agents that are substrates or inhibitors of Pgp. In particular, the flavonoids quercetin, apigenin, galangin and genistein, which are all structurally related to flavopiridol, have been shown to interact with Pgp, thereby modulating its transport capabilities and altering drug resistance (Critchfield et al, 1994; Castro and Altenberg, 1997; Conseil et al, 1998). The structural similarities between flavopiridol and these previously studied agents raise the possibility that flavopiridol might also be capable of interacting with Pgp.

Because of the potentially important implications of Pgp-mediated resistance in the clinical setting (Ling, 1997; Bradshaw and Arceci, 1998; Kaye, 1998; Volm, 1998), we have specifically investigated the effects of Pgp on flavopiridol-induced cell cycle arrest and cytotoxicity in $CH^RC5$ ($Pgp^{hi}$) and parental AuxB1 ($Pgp^{lo}$) Chinese hamster ovary cells. In previous studies, this pair of cell lines has been utilized not only to clone the Pgp cDNA and gene (Riordan et al, 1985; Van der Bliek et al, 1986), but also to investigate the role of Pgp in resistance to a wide variety of drugs (Bech-Hanson et al, 1976; Riordan et al, 1985; Hendricks et al, 1992). Compared to the AuxB1 cells, previous studies have demonstrated that $CH^RC5$ cells are 30-, 25-, 40-and 300-fold resistant to etoposide, doxorubicin, vinblastine, and colchicine, respectively (Bech-Hanson et al, 1976; Hendricks et al, 1992). The present studies indicate that the effects of flavopiridol are attenuated in cells that overexpress Pgp, but the degree of resistance is much lower than that observed with other agents.

### MATERIALS AND METHODS

### Materials

Flavopiridol was provided by the Pharmaceutical Resources Branch of the National Cancer Institute (Bethesda). Paclitaxel, cisplatin, propidium iodide, quinidine, and verapamil were

*Received 3 August 2000*
*Revised 13 December 2000*
*Accepted 15 December 2000*

*Correspondence to:* KC Bible; E-mail: bible.keith@mayo.edu purchased from Sigma (St. Louis). Stock solutions of flavopiridol, paclitaxel, quinidine and verapamil were prepared in DMSO and stored at –20˚C. Cisplatin stocks were prepared in DMSO immediately before use. Other reagents were obtained as previously described (Hendricks et al, 1992; Bible and Kaufmann, 1997).

### Cell lines

CH$^R$C5 Chinese hamster cells (Bech-Hanson et al, 1976), which contain an amplified mdr1 gene (Van der Bliek et al, 1986) and express at least 10-fold more Pgp than parental AuxB1 cells (Kartner et al, 1985; Hendricks et al, 1992) were kindly provided by Dr Victor Ling (British Columbia Cancer Research Center, Vancouver). These cells were cultured in medium A (α-MEM containing ribonucleotides and deoxyribonucleotides, 10% heat-inactivated fetal bovine serum, 100 units ml$^{-1}$ penicillin G, 100 µg ml$^{-1}$ streptomycin and 2 mM glutamine). Cells maintained under subconfluent conditions at 37˚C in an atmosphere of humidified 5% (w/w) $CO_2$ were passaged twice weekly.

Colony-forming assays were performed as previously described (Bible and Kaufmann, 1997). In brief, 1000 AuxB1 or CH$^R$C5 cells were seeded in triplicate in 35 mm tissue culture dishes containing 2 ml of medium A. After a 12–16 h incubation to allow cells to adhere, drugs were added to the indicated final concentrations from 1000-fold concentrated stocks. After a 24-h incubation, plates were washed twice with serum-free medium and incubated in medium A for an additional 6–7 days to allow colonies to form. Colonies stained with Coomassie brilliant blue were manually counted as previously described (Hendricks et al, 1992; Bible and Kaufmann, 1997). Where indicated, quinidine or verapamil (10 µM) was added 5–10 min prior to flavopiridol or paclitaxel. Neither quinidine or verapamil alone altered colony formation in CH$^R$C5 or AuxB$_1$ cells.

### Flow cytometry

Aliquots containing ~$1 \times 10^6$ cells in 100 mm tissue culture dishes were treated with flavopiridol as described above and harvested by trypsinization. All further steps were performed at 4˚C unless otherwise indicated. Cells were washed in calcium- and magnesium-free phosphate buffered saline (PBS), resuspended in 300 µl PBS, and fixed by addition of an equal volume of 95% ethanol. Cells were then washed twice with PBS, digested with RNase A, and stained with 50 µg ml$^{-1}$ propidium iodide as described (Bible, 1997). Samples were examined using a Becton Dickinson FACScan (San Jose) using an excitation wavelength of 488 nm and an emission wavelength of 585 ± 21 nm. Data were analysed using ModFit software (Verity Software, Topsham) or PC-LYSIS (Becton Dickinson).

### Assessment of cell viability

In order to directly assess cell viability, AuxB1 and CH$^R$C5 cells were grown to 30% confluence in 100 mm dishes and treated with varying concentrations of flavopiridol for 24 h. At the end of the incubation, adherent cells were released by trypsinization and combined with cells in the culture supernatant before cell viability was assessed using trypan blue as previously described (Bible and Kaufmann, 1996).

### Affinity labelling of P-glycoprotein in membrane vesicles

The ability of flavopiridol to interact with the drug-binding site of Pgp was assessed by affinity labelling (modified from Safa et al, 1987; Yang et al, 1989, as previously described in Sha et al, 1996). Membrane vesicles were prepared from CH$^R$C5 cells by the method of Lever (1977) and stored in 1 ml aliquots of buffer B (250 mM sucrose containing 10 mM Tris-HCl, pH 7.5 at 20˚C) at –70˚C for up to 2 months. Aliquots containing 100 µg of membrane protein (estimated by the method of Bradford, 1976) were incubated for 60 min at 22˚C in the dark with 50 nM [$^3$H]-azidopine in the absence or presence of 100 µM quinidine or various concentrations of flavopiridol. The samples were then placed on ice and irradiated for 15 min at a distance of 10 cm from a 10 W germicidal ultraviolet light (Yang et al, 1989).

The irradiated vesicles were recovered by ultracentrifugation and solubilized in SDS sample buffer at room temperature (Greenberger et al, 1988). Aliquots containing 40 µg of protein were subjected to SDS-PAGE on 5–15% (w/v) acrylamide gels. After staining with Coomassie blue to confirm equivalent recovery of the samples, gels were impregnated with Amplify (Amersham, Arlington Heights) according to the manufacturer's instructions, and subjected to fluorography using preflashed Kodak Xomat AR-5 film and appropriate intensifying screens.

### Statistics

Reliabilities of differences in sample means (statistical significances) were calculated using the two-tailed Student's *t*-tests and pooled estimates of sample variances.

### RESULTS

### Effects of P-glycoprotein on colony formation

In order to examine the potential effects of Pgp on flavopiridol-induced cytotoxicity, AuxB1 and CH$^R$C5 cells were exposed to varying concentrations of flavopiridol for 24 h and allowed to form colonies under drug-free conditions. To provide a basis for comparison, the cells were also exposed to paclitaxel, a well-characterized Pgp substrate (Greenberger et al, 1988; Bhalla et al, 1994), or cisplatin, which is not transported by Pgp. Results of this analysis (Figure 1A) revealed that the $IC_{50}$ for flavopiridol was 90.2 ± 6.6 nM in AuxB1 cells and 117.3 ± 2.3 nM in CH$^R$C5 cells ($n = 4$, $P < 0.01$), indicating a requirement for 30% higher extracellular drug concentrations to achieve the same effect in the Pgp$^{hi}$ CH$^R$C5 cell line. In comparison, the CH$^R$C5 cells required 6.2 ± 1.3-fold more paclitaxel (Figure 1B) and 20-fold more etoposide (Hendricks et al, 1992) than the AuxB1 cells while demonstrating no resistance to cisplatin (Figure 1C).

### Effects of P-glycoprotein modulators on flavopiridol-induced cytotoxicity

In order to determine whether the observed differences between the two clones reflected clonal variation as opposed to a bona fide effect of Pgp, we examined the effects of Pgp modulators on flavopiridol sensitivity. Of the various modulators that have been identified (Ford et al, 1996), we focused on verapamil and quinidine because of the widespread availability of these agents and the

© 2001 Cancer Research Campaign

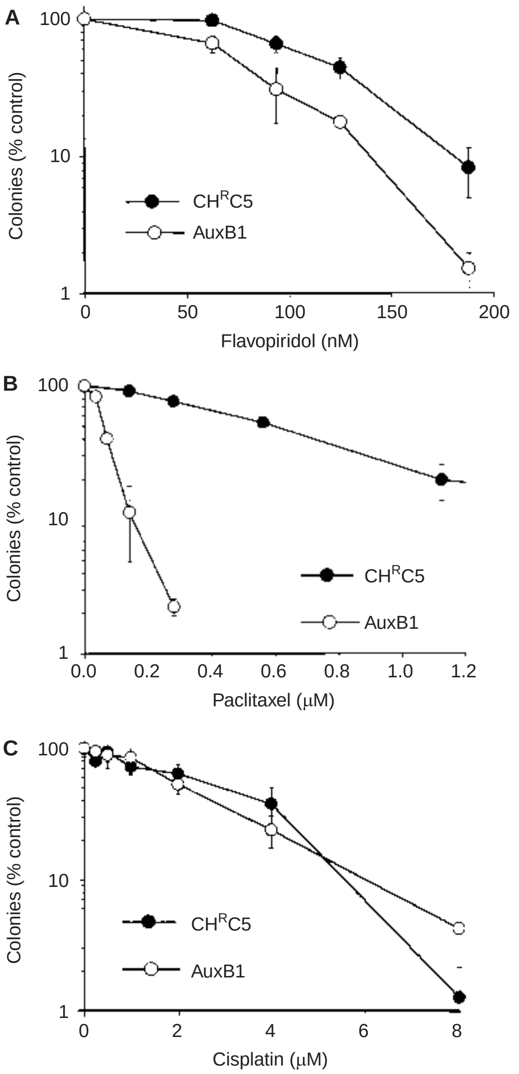

**Figure 1** Effects of P-glycoprotein on sensitivities to (**A**) flavopiridol, (**B**), paclitaxel and (**C**) cisplatin in parental AuxB1 (Pgp$^{lo}$) and colchicine-selected CH$^{R}$C5 (Pgp$^{hi}$) cells. Error bars represent ± 1 sample standard deviation (triplicate plates). Cells were exposed to flavopiridol for 24 h and then cultured in drug-free medium for 7 days prior to assessing colony formation. Results are representative of four independent experiments

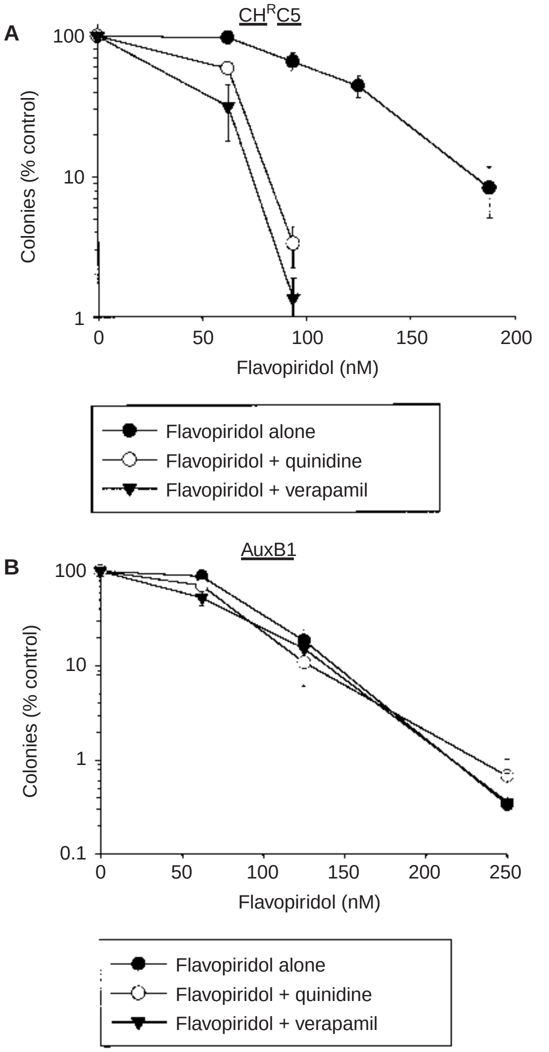

**Figure 2** Effects of P-glycoprotein modulators on flavopiridol-induced cytotoxicity in (**A**) CH$^{R}$C5 (Pgp$^{hi}$) and (**B**) AuxB1 (Pgp$^{lo}$) cells. Error bars represent ± 1 sample standard deviation (triplicate plates). Cells were exposed to flavopiridol for 24 h and then cultured in drug-free medium for 7 days prior to assessing colony formation. Results are representative of four independent experiments reported selectivity of quinidine for Pgp as opposed to other ABC cassette transporters (Willingham et al, 1986; Cole et al, 1989). As illustrated in Figure 2A, treatment of the CH$^{R}$C5 (Pgp$^{hi}$) cells with either quinidine or verapamil enhanced the ability of flavopiridol to inhibit colony formation. Similar effects, albeit of greater magnitude, were observed with paclitaxel (data not shown). In contrast, quinidine and verapamil had little effect on flavopiridol sensitivity in the AuxB1 (Pgp$^{lo}$) cell line (Figure 2B).

### Effect of P-glycoprotein on flavopiridol-induced cell cycle arrest and cytotoxicity

Because of concern that colony formation assays might not accurately reflect cytotoxicity (Waldman et al, 1997), further experiments examined the effects of flavopiridol using different assays. Previous studies (Kaur et al, 1992; Carlson et al, 1996; Bible and Kaufmann, 1997) have demonstrated that flavopiridol induces arrest in the $G_1$ and $G_2$ phases of the cell cycle. For cell lines that contain a predominance of $G_1$ cells, this cell cycle arrest is most reliably observed by examining the size of the $G_2$ population.

When the cell cycle effects of flavopiridol on AuxB1 and CH$^{R}$C5 cells were assessed by flow cytometry, fewer of the CH$^{R}$C5 cells arrested in $G_2$ at each drug concentration (Figure 3A). The same analysis revealed that CH$^{R}$C5 cultures contained fewer cells with subdiploid DNA content (a hallmark of apoptosis) after flavopiridol treatment (Figure 3B), raising the possibility that the Pgp$^{hi}$ cell line was potentially resistant to the cytotoxic as well as cell cycle effects of flavopiridol. To further evaluate this possibility, AuxB1 and CH$^{R}$C5 cells were exposed to varying concentrations of flavopiridol for 24 h and examined for ability to exclude trypan blue. Results of this analysis (Figure 3C) indicated that CH$^{R}$C5 cells were resistant to the cytotoxic effects of flavopiridol, in agreement with the results obtained with the other methods.

© 2001 Cancer Research Campaign

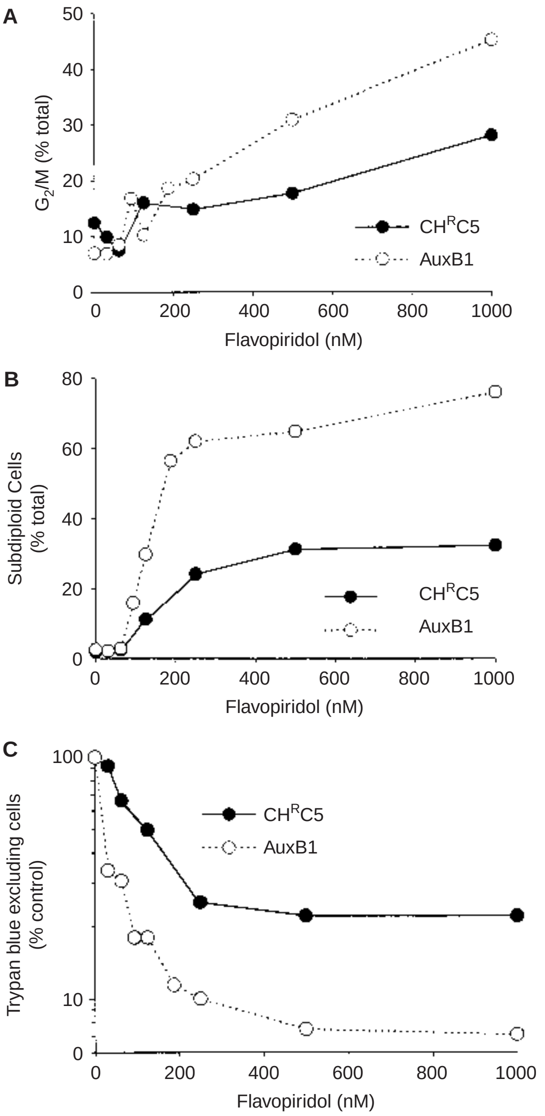

**Figure 3** Effects of P-glycoprotein on flavopiridol-induced alterations in cell cycle distribution and cytotoxicity in CH$^R$C5 (Pgp$^{hi}$) and AuxB1 (Pgp$^{lo}$) cells. (**A**) Effects of Pgp on flavopiridol-induced accumulation of cells in the $G_2$/M phase of the cell cycle as assessed by flow cytometry. (**B**) Effects of Pgp on flavopiridol-induced accumulation of subdiploid cells as assessed by flow cytometry. (**C**) Effects of Pgp on flavopiridol-induced cytotoxicity as assessed by trypan blue staining. Results in each panel are representative of three independent experiments

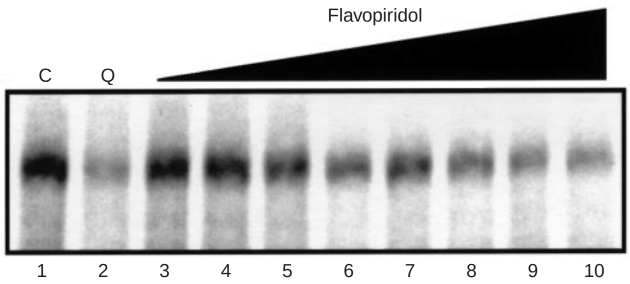

**Figure 4** Effects of quinidine or flavopiridol on the covalent binding of [$^3$H]-azidopine to P-glycoprotein in isolated membrane vesicles. Competitors added with azidopine were vehicle control (C, lane 1), 100 μM quinidine (Q, lane 2), or flavopiridol at 0.16, 0.32, 0.63, 1.25, 2.5, 5, 10 or 20 μM flavopiridol (lanes 3–10, respectively). Results are representative of three independent experiments

### Affinity labelling

The interaction between Pgp and various substrates or modulators has been studied under cell-free conditions using the photoactivatable substrate azidopine (Safa et al, 1987). In these experiments, agents that bind to the same site as azidopine decrease the amount of azidopine covalently bound to Pgp after photoactivation. To determine whether flavopiridol was directly binding to Pgp, CH$^R$C5 membrane vesicles were incubated with [$^3$H]-azidopine in the absence or presence of flavopiridol, then subjected to ultraviolet light to crosslink the azidopine to Pgp. Quinidine served as a positive control. As shown in Figure 4, quinidine markedly inhibited the binding of [$^3$H]-azidopine to Pgp in comparison to vehicle control (Figure 4, lanes 2 and 1, respectively). Flavopiridol also inhibited [$^3$H]-azidopine binding to Pgp in a dose-dependent manner, although the extent of inhibition at the highest flavopiridol concentration tested (20 μM) was lower than the inhibition by 100 μM quinidine (lanes 10 and 2, Figure 4).

### DISCUSSION

Drug resistance is a major impediment to the successful treatment of cancer. Despite its novel structure and mechanism of action, flavopiridol causes tumour regression in only a minority of treated patients (Senderowicz et al, 1998), suggesting that resistance to this agent might also be a problem in the clinical setting. Previous studies have not only demonstrated that Pgp can be expressed in a wide variety of tumour types (Ling, 1997; Bradshaw and Arceci, 1998; Kaye, 1998), but have also indicated that other flavone derivatives can directly interact with this transporter (Critchfield et al, 1994; Castro and Altenbog, 1997; Conseil et al, 1998). Based on these considerations, we have examined the effects of Pgp on flavopiridol-induced cell cycle arrest and cytotoxicity. Results of these analyses have potential implications for future clinical development of this agent.

The present observations indicate that flavopiridol is less active in cells that overexpress Pgp. This effect of Pgp is manifest as a decrease in cell cycle arrest (Figure 3A), as well as diminished cytotoxicity as assessed by three different assays (Figures 1A, 3B and 3C). Consistent with these results, we observed that flavopiridol inhibits the binding of the affinity label azidopine to Pgp in membrane vesicles in vitro (Figure 4) and Pgp modulators enhance the effect of flavopiridol in cells that overexpress Pgp (Figure 2A). All of these findings are consistent with an interaction between flavopiridol and Pgp.

The present data do not rule out the possibility that flavopiridol, acting as a kinase inhibitor, might also alter Pgp phosphorylation and function. Such a model, however, would not explain the effect of Pgp inhibitors on flavopiridol action (Figure 2) or the effect of flavopiridol on ($^3$H)-azidopine binding under cell-free conditions (Figure 4). Instead, these observations are best explained by a direct interaction between flavopiridol and Pgp. On the other hand, comparison of the data in Figures 1 and 4 indicates that higher flavopiridol concentrations are required to displace azidopine from Pgp than are required to kill cells. This disparity raises the possibility that Pgp might be affecting flavopiridol sensitivity in some indirect manner at the low flavopiridol concentrations used in the cytotoxicity assays. We note, however, that a similar requirement for high drug concentrations has been observed when other Pgp substrates, including paclitaxel, doxorubicin and colchicine, have been used to displace affinity labels from Pgp (Greenberger et al,

© 2001 Cancer Research Campaign

1990). Thus, it is more likely that higher flavopiridol concentrations required to prevent [$^3$H]-azidopine labelling reflect the artificial conditions of the photolabelling experiment, i.e. the need to completely prevent all noncovalent binding of azidopine to Pgp during the entire period of illumination in order to see a decrease in the covalently bound label.

It is also important to note that the effects of Pgp on the action of flavopiridol are much smaller than effects on other anticancer drugs. The relative resistance of $CH^RC5$ cells (i.e. the ratio of $IC_{50}$s of $CH^RC5$ cells compared to parental AuxB1 cells) is ~ 20 for etoposide, 30 for doxorubicin (Hendricks et al, 1992) and 6 for paclitaxel (Figure 1B), but only 1.3 for flavopiridol. While it would be potentially possible to make the effects of Pgp on flavopiridol appear more dramatic, e.g. by picking a cell line that expresses more Pgp and is 10 000 fold resistant to doxorubicin, the fact that the flavopiridol-Pgp interaction is a weak one (Figure 4) would still remain.

The realization that flavopiridol is a Pgp substrate raises the possibility that malignancies such as renal cell carcinoma, colon cancer and pancreatic cancer, which universally express Pgp (Goldstein et al, 1989), might have some degree of de novo flavopiridol resistance on this basis. The significance, however, of the relatively low level of flavopiridol resistance imparted by Pgp overexpression remains to be established, particularly in the clinical setting. Although the present study appears to identify one potential mechanism of flavopiridol resistance, other mechanisms also undoubtedly exist, as illustrated by the recently characterized pair of ovarian cell lines studied in our laboratory (Bible et al, 2000).

### ACKNOWLEDGEMENTS

This work was supported in part by grants from the National Cancer Institute U01 CA69912-4, the American Cancer Society RPG CCE-98842, and the Jack Taylor Family Foundation. The authors greatly acknowledge a gift of flavopiridol from Edward Sausville, advice of James Tarara regarding performance and analysis of flow cytometry, assistance of Jen Davis with the affinity labeling experiments and secretarial assistance of Deb Strauss.

### REFERENCES

Arguello F, Alexander M, Sterry JA, Tudor G, Smith EM, Kalavar NT, Greene JF Jr, Koss W, Morgan CD, Stinson SF, Siford TJ, Alvord WG, Klabansky RL and Sausville EA (1998) Flavopiridol induces apoptosis of normal lymphoid cells, causes immunosuppression, and has potent antitumor activity *in vivo* against human leukemia and lymphoma xenografts. *Blood* **91**: 2482–2490

Bech-Hanson NT, Till JE and Ling V (1976) Pleiotropic phenotype of colchicine-resistant CHO cells: cross-resistance and collateral sensitivity. *J Cell Physiol* **88**: 23–31

Bhalla K, Huang Y, Tang C, Self S, Ray S, Mahoney ME, Ponnathpur V, Tourkina E, Ibrado AM and Bullock G (1994) Characterization of a human myeloid leukemia cell line highly resistant to taxol. *Leukemia* **8**: 465–475

Bible KC and Kaufmann SH (1996) Flavopiridol (NSC 649890, L86-8275): a cytotoxic flavone that induces cell death in human lung carcinoma cells. *Cancer Res* **56**: 4856–4861

Bible KC and Kaufmann SH (1997) Cytotoxic synergy between flavopiridol (NSC 649890, L86-8275) and various antineoplastic agents: importance of sequence of administration. *Cancer Res* **57**: 3375–3380

Bible KC, Boerner SA, Kirkland K, Anderl KL, Duane Bartelt J, Svingen PA, Kottke TJ, Eckdahl S, Stalboerger PG, Jenkins RB and Kaufmann SH (2000) Characterization of an ovarian carcinoma cell line resistant to cisplatin and flavopiridol. *Clin Cancer Res* **6**: 661–670

Bradford M (1976) A rapid and sensitive method for the quantitation of microgram quantities of protein utilizing the principle of protein-dye binding. *Anal Biochem* **72**: 248

Bradshaw DM and Arceci RJ (1998) Clinical relevance of transmembrane drug efflux as a mechanism of multidrug resistance. *J Clin Oncol* **16**: 3674–3690

Brüsselbach S, Nettelbeck DM, Sedlacek H-H and Müller R (1998) Cell cycle-independent induction of apoptosis by the anti-tumor drug flavopiridol in endothelial cells. *Int J Cancer* **77**: 146–152

Byrd JC, Shinn C, Waselenko JK, Fuchs EJ, Lehman TA, Nguyen PL, Flinn IW, Diehl LF, Sausville E and Grever MR (1998) Flavopiridol induces apoptosis in chronic lymphocytic leukemia cells via activation of caspase-3 without evidence of bcl-2 modulation or dependence upon functional p53. *Blood* **92**: 3804–3816

Carlson BA, Dubay MM, Sausville EA, Brizuela L and Worland PJ (1996) Flavopiridol induces $G_1$ arrest with inhibition of cyclin-dependent kinase (CDK) 2 and CDK4 in human breast carcinoma cells. *Cancer Res* **56**: 2973–2978

Castro AF and Altenberg GA (1997) Inhibition of drug transport by genistein in multidrug-resistant cells expressing P-glycoprotein. *Biochem Pharmacol* **53**: 89–93

Cole SP, Downes HF and Slovak ML (1989) Effect of calcium antagonists on the chemosensitivity of two multidrug-resistant human tumour cell lines which do not overexpress P-glycoprotein. *Br J Cancer* **59**: 42–46

Conseil G, Baubichon-Cortay H, Dayan G, Jault JM, Barron D and Di Pietro A (1998) Flavonoids: a class of modulators with bifunctional interactions at vicinal ATP- and steroid-binding sites on mouse P-glycoprotein. *Proc Natl Acad Sci USA* **95**: 9831–9836

Critchfield JW, Welsh CJ, Phang JM and Yeh GC (1994) Modulation of adriamycin accumulation and efflux by flavonoids in HCT-15 colon cells. Activation of P-glycoprotein as a putative mechanism. *Biochem Pharmacol* **48**: 1437–1445

Czech J, Hoffmann D, Naik R and Sedlacek H-H (1995) Antitumoral activity of flavone L86-8275. *Int J Oncol* **6**: 31–36

de Azevedo WF, Mueller-Dieckmann H-J, Schulze-Gahmen U, Worland PJ, Sausville E and Kim S-H (1996) Structural basis for specificity and potency of a flavonoid inhibitor of human CDK2, a cell cycle kinase. *Proc Natl Acad Sci USA* **93**: 2735–2740

Dress M, Dengler WA, Roth T, Labonte H, Mayo J, Malspeis L, Grever M, Sausville EA and Fiebig HH (1997) Flavopiridol (L86-8275): Selective antitumor in vitro and activity in vivo for prostate carcinoma cells. *Clin Cancer Res* **3**: 273–279

Ford JM, Yang JM and Hait WN (1996) P-glycoprotein-mediated multidrug resistance: experimental and clinical strategies for its reversal. *Cancer Treat Res* **87**: 3–38

Goldstein LJ, Galski H, Fojo A, Willingham M, Lai S-L, Gazdar A, Pirker R, Green A, Crist W, Brodear GM, Lieber M, Cossman J, Gottesman MM and Pastan I (1989) Expression of a multidrug resistance gene in human cancers. *J Natl Cancer Inst* **81**: 116–124

Greenberger LM, Williams SS, Georges E, Ling V and Horwitz SB (1988) Electrophoretic analysis of P-glycoproteins produced by mouse J774.2 and Chinese hamster ovary multidrug-resistant cells. *J Natl Cancer Inst* **80**: 506

Greenberger LM, Huang Yang C-P, Gindin E and Band Horwitz S (1990) Photoaffinity probes for the $\alpha_1$-adrenergic receptor and the calcium channel bind to a common domain in P-glycoprotein. *J Biol Chem* **265**: 4394–4401

Hendricks CB, Rowinsky EK, Grochow LB, Donehower RC and Kaufmann SH (1992) Effect of P-glycoprotein expression on the accumulation and cytotoxicity of topotecan (SK&F 104864), a new camptothecin analogue. *Cancer Res* **52**: 2268–2278

Kartner N, Evernden-Porelle D, Bradley G and Ling V (1985) Detection of P-glycoprotein in multidrug-resistant cell lines by monoclonal antibodies. *Nature* **316**: 820–823

Kaur G, Stetler-Stevenson M, Sebers S, Worland P, Sedlacek H, Myers C, Czech J, Naik R and Sausville E (1992) Growth inhibition with reversible cell cycle arrest of carcinoma cells by flavone L86-8275. *J Natl Cancer Inst* **84**: 1736–1740

Kaye SB (1998) Multidrug resistance: clinical relevance in solid tumours and strategies for circumvention. *Curr Opin Oncol* 10 Suppl: S15–S19

König A, Schwartz GK, Mohammad RM, A1-Katib A and Gabrilove JL (1997) The novel cyclin-dependent kinase inhibitor flavopiridol downregulates Bcl-2 and induces growth arrest and apoptosis in chronic B-cell leukemia lines. *Blood* **90**: 4307–4312

Lever J (1977) Active amino acid transport in plasma membrane vesicles from simian virus 40-transformed mouse fibroblasts. Characteristics of electrochemical $Na^+$ gradient-stimulated uptake. *J Biol Chem* **252**: 1990

© 2001 Cancer Research Campaign

Ling V (1997) Multidrug resistance: molecular mechanisms and clinical relevance. *Cancer Chemother Pharmacol* **40**: S3–S8
Losiewicz MD, Carlson BA, Kaur G, Sausville EA and Worland PJ (1994) Potent inhibition of CDC2 kinase activity by the flavonoid L86-8275. *Biochem Biophys Res Commun* **201**: 589–595
Parker BW, Kaur G, Nieves-Neira W, Taimi M, Kohlhagen G, Shimizu T, Losiewicz MD, Pommier Y, Sausville EA and Senderowicz AM (1998) Early induction of apoptosis in hematopoietic cell lines after exposure to flavopiridol. *Blood* **91**: 458–465
Patel V, Senderowicz AM, Pinto D, Jr., Igishi T, Raffeld M, Quintanilla-Martinez L, Ensley JF, Sausville EA and Gutkind JS (1998) Flavopiridol, a novel cyclin dependent kinase inhibitor, suppresses the growth of squamous cell carcinomas of the head and neck by inducing apoptosis. *J Clin Invest* **102**: 1674–1681
Riordan JR, Deuchars K, Kartner N, Alon N, Trent J and Ling V (1985) Amplification of P-glycoprotein genes in multidrug-resistant mammalian cell lines. *Nature* **316**: 817–819
Safa AR, Glover CJ, Sewell JL, Meyers MB, Biedler JL and Felsted RL (1987) Identification of the multidrug resistance-related membrane glycoprotein as an acceptor for calcium channel blockers. *J Biol Chem* **262**: 7884
Schlegel S, Klimecki W and List AF (1999) Breast cancer resistance protein (BCRP)-associated drug export and p53 inactivation impact flavopiridol (FLA) antitumor activity. *Proc Am Assoc Cancer Res* **40**: 669
Schwartz GK, Farsi K, Maslak P, Kelsen DP and Spriggs D (1997) Potentiation of apoptosis by flavopiridol in mitomycin-C-treated gastric and breast cancer cells. *Clin Cancer Res* **3**: 1467–1472
Sedlacek HH, Czech J, Naik R, Kaur G, Worland P, Losiewicz M, Parker B, Carlson B, Smith A, Senderowicz A and Sausville E (1996) Flavopiridol (L86 8275; NSC 649890), a new kinase inhibitor for tumor therapy. *Int J Oncol* **9**: 1143–1168
Senderowicz AM, Headlee D, Stinson SF, Lush RM, Kalil N, Villalba L, Hill K, Steinberg SM, Figg WD, Tompkins A, Arbuck SG and Sausville EA (1998) Phase I trial of continuous infusion flavopiridol, a novel cyclin-dependent kinase inhibitor, in patients with refractory neoplasms. *J Clin Oncol* **16**: 2986–2999
Sha EC, Sha MC and Kaufmann SH (1996) Evaluation of 2,6-diamino-N-([1-oxotridecyl)-2-piperidinyl)methyl)-hexanamide (NPC 15437), a protein kinase C inhibitor, as a modulator of P-glycoprotein-mediated in vitro. *Invest New Drugs* **13**: 285–294
Van der Bliek AM, Van der Velde-Koerts T, Ling V and Borst P (1986) Overexpression and amplification of five genes in a multidrug-resistant chinese hamster ovary cell line. *Mol Cell Biol* **6**: 1671–1678
Volm M (1998) Multidrug resistance and its reversal. *Anticancer Res* **18**: 2905–2917
Waldman T, Zhang Y, Dillehay L, Yu J, Kinzler K, Vogelstein B and Williams J (1997) Cell-cycle arrest versus cell death in cancer therapy. *Nature Med* **3**: 1034–1036
Willingham MC, Cornwell MM, Cardarelli CO, Gottesman MM and Pastan I (1986) Single cell analysis of daunomycin uptake and efflux in multi-drug-resistant and -sensitive KB cells: effects of verapamil and other drugs. *Cancer Res* **46**: 5941–5946
Wright J, Blatner GL and Cheson BD (1998) Clinical trials of flavopiridol. *Oncology* **12**: 1018–1024
Yang C-P, DePinho SG, Greenberger LM, Arceci RJ and Horwitz SB (1989) Progesterone interacts with P-glycoprotein in multidrug-resistant cells and in the endometrium of gravid uterus. *J Biol Chem* **264**: 782

*© 2001 Cancer Research Campaign*

## References

[PDF_00523] Arguello F., Alexander M., Sterry J. A., Tudor G., Smith E. M., Kalavar N. T., Greene J. F., Koss W., Morgan C. D., Stinson S. F. (1998). Flavopiridol induces apoptosis of normal lymphoid cells, causes immunosuppression, and has potent antitumor activity In vivo against human leukemia and lymphoma xenografts.. Blood.

[PDF_00528] Bech-Hansen N. T., Till J. E., Ling V. (1976). Pleiotropic phenotype of colchicine-resistant CHO cells: cross-resistance and collateral sensitivity.. J Cell Physiol.

[PDF_00531] Bhalla K., Huang Y., Tang C., Self S., Ray S., Mahoney M. E., Ponnathpur V., Tourkina E., Ibrado A. M., Bullock G. (1994). Characterization of a human myeloid leukemia cell line highly resistant to taxol.. Leukemia.

[PDF_00540] Bible K. C., Boerner S. A., Kirkland K., Anderl K. L., Bartelt D., Svingen P. A., Kottke T. J., Lee Y. K., Eckdahl S., Stalboerger P. G. (2000). Characterization of an ovarian carcinoma cell line resistant to cisplatin and flavopiridol.. Clin Cancer Res.

[PDF_00537] Bible K. C., Kaufmann S. H. (1997). Cytotoxic synergy between flavopiridol (NSC 649890, L86-8275) and various antineoplastic agents: the importance of sequence of administration.. Cancer Res.

[PDF_00534] Bible K. C., Kaufmann S. H. (1996). Flavopiridol: a cytotoxic flavone that induces cell death in noncycling A549 human lung carcinoma cells.. Cancer Res.

[PDF_00544] Bradford M. M. (1976). A rapid and sensitive method for the quantitation of microgram quantities of protein utilizing the principle of protein-dye binding.. Anal Biochem.

[PDF_00547] Bradshaw D. M., Arceci R. J. (1998). Clinical relevance of transmembrane drug efflux as a mechanism of multidrug resistance.. J Clin Oncol.

[PDF_00549] Brüsselbach S., Nettelbeck D. M., Sedlacek H. H., Müller R. (1998). Cell cycle-independent induction of apoptosis by the anti-tumor drug Flavopiridol in endothelial cells.. Int J Cancer.

[PDF_00552] Byrd J. C., Shinn C., Waselenko J. K., Fuchs E. J., Lehman T. A., Nguyen P. L., Flinn I. W., Diehl L. F., Sausville E., Grever M. R. (1998). Flavopiridol induces apoptosis in chronic lymphocytic leukemia cells via activation of caspase-3 without evidence of bcl-2 modulation or dependence on functional p53.. Blood.

[PDF_00557] Carlson B. A., Dubay M. M., Sausville E. A., Brizuela L., Worland P. J. (1996). Flavopiridol induces G1 arrest with inhibition of cyclin-dependent kinase (CDK) 2 and CDK4 in human breast carcinoma cells.. Cancer Res.

[PDF_00562] Castro A. F., Altenberg G. A. (1997). Inhibition of drug transport by genistein in multidrug-resistant cells expressing P-glycoprotein.. Biochem Pharmacol.

[PDF_00565] Cole S. P., Downes H. F., Slovak M. L. (1989). Effect of calcium antagonists on the chemosensitivity of two multidrug-resistant human tumour cell lines which do not overexpress P-glycoprotein.. Br J Cancer.

[PDF_00568] Conseil G., Baubichon-Cortay H., Dayan G., Jault J. M., Barron D., Di Pietro A. (1998). Flavonoids: a class of modulators with bifunctional interactions at vicinal ATP- and steroid-binding sites on mouse P-glycoprotein.. Proc Natl Acad Sci U S A.

[PDF_00572] Critchfield J. W., Welsh C. J., Phang J. M., Yeh G. C. (1994). Modulation of adriamycin accumulation and efflux by flavonoids in HCT-15 colon cells. Activation of P-glycoprotein as a putative mechanism.. Biochem Pharmacol.

[PDF_00577] De Azevedo W. F., Mueller-Dieckmann H. J., Schulze-Gahmen U., Worland P. J., Sausville E., Kim S. H. (1996). Structural basis for specificity and potency of a flavonoid inhibitor of human CDK2, a cell cycle kinase.. Proc Natl Acad Sci U S A.

[PDF_00581] Drees M., Dengler W. A., Roth T., Labonte H., Mayo J., Malspeis L., Grever M., Sausville E. A., Fiebig H. H. (1997). Flavopiridol (L86-8275): selective antitumor activity in vitro and activity in vivo for prostate carcinoma cells.. Clin Cancer Res.

[PDF_00585] Ford J. M., Yang J. M., Hait W. N. (1996). P-glycoprotein-mediated multidrug resistance: experimental and clinical strategies for its reversal.. Cancer Treat Res.

[PDF_00588] Goldstein L. J., Galski H., Fojo A., Willingham M., Lai S. L., Gazdar A., Pirker R., Green A., Crist W., Brodeur G. M. (1989). Expression of a multidrug resistance gene in human cancers.. J Natl Cancer Inst.

[PDF_00592] Greenberger L. M., Williams S. S., Georges E., Ling V., Horwitz S. B. (1988). Electrophoretic analysis of P-glycoproteins produced by mouse J774.2 and Chinese hamster ovary multidrug-resistant cells.. J Natl Cancer Inst.

[PDF_00595] Greenberger L. M., Yang C. P., Gindin E., Horwitz S. B. (1990). Photoaffinity probes for the alpha 1-adrenergic receptor and the calcium channel bind to a common domain in P-glycoprotein.. J Biol Chem.

[PDF_00599] Hendricks C. B., Rowinsky E. K., Grochow L. B., Donehower R. C., Kaufmann S. H. (1992). Effect of P-glycoprotein expression on the accumulation and cytotoxicity of topotecan (SK&F 104864), a new camptothecin analogue.. Cancer Res.

[PDF_00603] Kartner N., Evernden-Porelle D., Bradley G., Ling V. Detection of P-glycoprotein in multidrug-resistant cell lines by monoclonal antibodies.. Nature.

[PDF_00606] Kaur G., Stetler-Stevenson M., Sebers S., Worland P., Sedlacek H., Myers C., Czech J., Naik R., Sausville E. (1992). Growth inhibition with reversible cell cycle arrest of carcinoma cells by flavone L86-8275.. J Natl Cancer Inst.

[PDF_00610] Kaye S. B. (1998). Multidrug resistance: clinical relevance in solid tumours and strategies for circumvention.. Curr Opin Oncol.

[PDF_00612] König A., Schwartz G. K., Mohammad R. M., Al-Katib A., Gabrilove J. L. (1997). The novel cyclin-dependent kinase inhibitor flavopiridol downregulates Bcl-2 and induces growth arrest and apoptosis in chronic B-cell leukemia lines.. Blood.

[PDF_00616] Lever J. E. (1977). Active amino acid transport in plasma membrane vesicles from Simian virus 40-transformed mouse fibroblasts. Characteristics of electrochemical Na+ gradient-stimulated uptake.. J Biol Chem.

[PDF_00623] Ling V. (1997). Multidrug resistance: molecular mechanisms and clinical relevance.. Cancer Chemother Pharmacol.

[PDF_00625] Losiewicz M. D., Carlson B. A., Kaur G., Sausville E. A., Worland P. J. (1994). Potent inhibition of CDC2 kinase activity by the flavonoid L86-8275.. Biochem Biophys Res Commun.

[PDF_00628] Parker B. W., Kaur G., Nieves-Neira W., Taimi M., Kohlhagen G., Shimizu T., Losiewicz M. D., Pommier Y., Sausville E. A., Senderowicz A. M. (1998). Early induction of apoptosis in hematopoietic cell lines after exposure to flavopiridol.. Blood.

[PDF_00633] Patel V., Senderowicz A. M., Pinto D., Igishi T., Raffeld M., Quintanilla-Martinez L., Ensley J. F., Sausville E. A., Gutkind J. S. (1998). Flavopiridol, a novel cyclin-dependent kinase inhibitor, suppresses the growth of head and neck squamous cell carcinomas by inducing apoptosis.. J Clin Invest.

[PDF_00637] Riordan J. R., Deuchars K., Kartner N., Alon N., Trent J., Ling V. Amplification of P-glycoprotein genes in multidrug-resistant mammalian cell lines.. Nature.

[PDF_00640] Safa A. R., Glover C. J., Sewell J. L., Meyers M. B., Biedler J. L., Felsted R. L. (1987). Identification of the multidrug resistance-related membrane glycoprotein as an acceptor for calcium channel blockers.. J Biol Chem.

[PDF_00646] Schwartz G. K., Farsi K., Maslak P., Kelsen D. P., Spriggs D. (1997). Potentiation of apoptosis by flavopiridol in mitomycin-C-treated gastric and breast cancer cells.. Clin Cancer Res.

[PDF_00653] Senderowicz A. M., Headlee D., Stinson S. F., Lush R. M., Kalil N., Villalba L., Hill K., Steinberg S. M., Figg W. D., Tompkins A. (1998). Phase I trial of continuous infusion flavopiridol, a novel cyclin-dependent kinase inhibitor, in patients with refractory neoplasms.. J Clin Oncol.

[PDF_00658] Sha E. C., Sha M. C., Kaufmann S. H. (1996). Evaluation of 2,6-diamino-N-([1-(1-oxotridecyl)-2-piperidinyl]methyl)- hexanamide (NPC 15437), a protein kinase C inhibitor, as a modulator of P-glycoprotein-mediated resistance in vitro.. Invest New Drugs.

[PDF_00662] Van der Bliek A. M., Van der Velde-Koerts T., Ling V., Borst P. (1986). Overexpression and amplification of five genes in a multidrug-resistant Chinese hamster ovary cell line.. Mol Cell Biol.

[PDF_00665] Volm M. (1998). Multidrug resistance and its reversal.. Anticancer Res.

[PDF_00666] Waldman T., Zhang Y., Dillehay L., Yu J., Kinzler K., Vogelstein B., Williams J. (1997). Cell-cycle arrest versus cell death in cancer therapy.. Nat Med.

[PDF_00669] Willingham M. C., Cornwell M. M., Cardarelli C. O., Gottesman M. M., Pastan I. (1986). Single cell analysis of daunomycin uptake and efflux in multidrug-resistant and -sensitive KB cells: effects of verapamil and other drugs.. Cancer Res.

[PDF_00673] Wright J., Blatner G. L., Cheson B. D. (1998). Clinical trials referral resource. Clinical trials of flavopiridol.. Oncology (Williston Park).

[PDF_00675] Yang C. P., DePinho S. G., Greenberger L. M., Arceci R. J., Horwitz S. B. (1989). Progesterone interacts with P-glycoprotein in multidrug-resistant cells and in the endometrium of gravid uterus.. J Biol Chem.

